# Tongxinluo May Alleviate Inflammation and Improve the Stability of Atherosclerotic Plaques by Changing the Intestinal Flora

**DOI:** 10.3389/fphar.2022.805266

**Published:** 2022-04-01

**Authors:** Yan Qi, Wenzhao Liu, Xuefang Yan, Chen Zhang, Chunmei Zhang, Lingxin Liu, Xuehui Zheng, Mengying Suo, Yun Ti, Mei Ni, Meng Zhang, Peili Bu

**Affiliations:** The Key Laboratory of Cardiovascular Remodeling and Function Research, Chinese Ministry of Education, Chinese National Health Commission and Chinese Academy of Medical Sciences, The State and Shandong Province Joint Key Laboratory of Translational Cardiovascular Medicine, Department of Cardiology, Qilu Hospital, Cheeloo College of Medicine, Shandong University, Jinan, China

**Keywords:** Tongxinluo, atherosclerotic plaque, NLRP3, intestinal flora, metabolism, trans-ferulic acid

## Abstract

Intestinal flora plays an important role in atherosclerosis. Tongxinluo, as a multi-target Chinese medicine to improve atherosclerosis, whether it can improve atherosclerosis by affecting the intestinal flora is worth exploring. We established a vulnerable plaque model of atherosclerosis in New Zealand white rabbits by high cholesterol diet and balloon injury (HCB), and performed Tongxinluo intervention. We detected the level of inflammation by immunohistochemistry, Western Blot, and ELISA, analyzed plaque characteristics by calculating the vulnerability index, and analyzed the changes of gut microbiota and metabolites by 16S rRNA gene sequencing and untargeted metabolomic sequencing. The results showed that Tongxinluo intervention improved plaque stability, reduced inflammatory response, inhibited NLRP3 inflammatory pathway, increased the relative abundance of beneficial bacteria such as *Alistipes* which reduced by HCB, and increased the content of beneficial metabolites such as trans-ferulic acid in feces. Through correlation analysis, we found that some metabolites were significantly correlated with some bacteria and some inflammatory factors. In particular, the metabolite trans-ferulic acid was also significantly positively correlated with plaque stability. Our further studies showed that trans-ferulic acid could also inhibit the NLRP3 inflammatory pathway. In conclusion, Tongxinluo can improve plaque stability and reduce inflammation in atherosclerotic rabbits, which may be achieved by modulating intestinal flora and intestinal metabolism. Our study provides new views for the role of Tongxinluo in improving atherosclerotic vulnerable plaque, which has important clinical significance.

## 1 Introduction

Atherosclerosis (AS) is a chronic disease of the arterial wall that results in cardiovascular diseases, including ischemic heart disease, ischemic stroke and peripheral artery disease (Herrington et al., 2016), and is the main cause of death and loss of productive life years worldwide (Libby et al., 2011). The pathogenesis of atherosclerosis is complex and includes chronic inflammation, oxidative stress, genetic susceptibility, immune disorders and epigenetics (Xu et al., 2019). However, there are relatively few treatments for AS at present, and the main clinical strategy is still lowering lipids. Inflammation is one of the classic and most common mechanisms of AS, and the improvement or inhibition of inflammation is essential to reduce cardiovascular events (Ridker et al., 2017; Vaidya et al., 2018). However, to date, no clinically applicable drugs can be specifically used to improve the inflammatory state of patients with an atherosclerotic disease.

Tongxinluo (TXL) in capsule form is a compound formulated according to the meridian theory of traditional Chinese medicine. Tongxinluo is a mixture of plant and animal products, including *Panax ginseng* C.A.Mey (Ren Shen), *Hirudo nipponica* Whitman (Shui Zhi), *Scolopendra subspinipes mutilans* L. Koch (Wu Gong), *Eupolyphaga sinensis* Walker (Tu Bie Chong), *Buthus martensii* Karsch (Quan Xie), *Cryptotympana pustulata* Fabricius (Chan Tui), *Paeonia lactiflora* Pall (Chi Shao), *Dryobalanops aromatica* C.F.Gaertn. (Bing Pian), *Santalum album* L (Tan Xiang), *Boswellia carterii* Birdw. (Ru Xiang), *Dalbergia odorifera* T.C.Chen (Jiang Xiang) and *Ziziphus jujuba* Mill (Suan Zao Ren) (Li et al., 2021), and was approved in 1996 by the State Food and Drug Administration of China for the treatment of angina pectoris and ischemic stroke. However, the mechanism by which Tongxinluo improves these atherosclerotic diseases is still unclear. Previous studies have suggested that Tongxinluo can exert its anti-inflammatory effects by reducing the expression of monocyte chemoattractant protein 1 (MCP-1) (Yao et al., 2014), NF-κB (Zhang et al., 2018)and others and increasing the stability of atherosclerotic vulnerable plaques (Zhang et al., 2009). NLRP3 is an important inflammatory factor involved in atherosclerosis. NLRP3 (NOD-like receptor family, pyrin domain containing 3) belongs to the nucleotide binding domain and leucine rich repeat (LRR) protein family (Ting et al., 2008) and can activate pro-caspase 1. Activated caspase 1 can cleave the precursors of interleukin -1β (IL-1β) and interleukin-18 (IL-18) into active forms (Mangan et al., 2018). As the main cytokine involved in inflammation, activated IL-1β increases the expression of a variety of pro-inflammatory factors. However, whether TXL exerts its anti-atherosclerotic effect by inhibiting the important NLRP3/Caspase-1/IL-1β inflammatory pathway has not yet been confirmed. Moreover, the specific mechanism by which TXL improves the stability of atherosclerotic plaques requires further study.

In recent years, the mechanisms by which the intestinal flora affect inflammation and atherosclerosis have become a hot research topic worldwide (Barrington and Lusis, 2017). In the past 10 years, the development of genome sequencing technology (especially 16S rRNA- and amplicon-based metagenomics), culture technology, and bioinformatics has led the intestinal flora, the “forgotten organ”, to receive increasing attention. The intestinal flora is mainly located in the colon and contains 10^13^∼10^14^ different kinds of bacteria. These bacteria encode unique genes, up to 100 times more genes than in the human genome (Ley et al., 2006; Qin et al., 2010), and can regulate the homeostasis of a variety of metabolic processes in the host and interfere with the host’s inflammatory state. Studies have confirmed that intestinal flora imbalances play important roles in many diseases, including atherosclerosis (Tang and Hazen, 2014), Parkinson’s disease (Pellegrini et al., 2018), allergies (Ramakrishnan and Frank, 2018), non-alcoholic fatty liver [NAFLD] (Alisi et al., 2012), type 2 diabetes (T2DM), obesity (Meijnikman et al., 2018) and many others. Studies of the relationship between the intestinal flora and atherosclerosis have shown that some bacteria and their related metabolites, such as TMAO, have atherogenic effects (Zhu et al., 2016), while other bacteria, such as *Faecalibacterium prausnitzii* and *Roseburia intestinalis*, and their metabolites, such as butyric acid, have anti-atherosclerotic effects. In short, current research suggests that the intestinal flora is involved in the regulation of atherosclerosis and that its regulatory mechanism is very complicated. NLRP3 (Yao et al., 2017; Singh et al., 2019) as an important mediator of atherosclerotic plaque formation, as well as its downstream factor IL-1β, were also closely related to the intestinal flora. As an oral Chinese medicine, Tongxinluo will inevitably encounter the intestinal flora. Whether Tongxinluo can inhibit the expression of NLRP3 and its related inflammatory factors by regulating the composition and metabolism of the intestinal flora and then exert its anti-atherosclerotic inflammatory effects and stabilize vulnerable plaques requires further exploration.

To clarify the relationship among Tongxinluo, vulnerable plaques, and the intestinal flora, we used high cholesterol feeding and balloon injury (HCB) technology to establish an atherosclerotic vulnerable plaque model in rabbits and then orally administered Tongxinluo to the model rabbits. The results of the experiments showed that TXL improved atherosclerosis, especially by reducing the expression of members of the vascular NLRP3 pathway and enhancing the stability of plaques. These effects were strongly related to the ability of TXL to regulate the intestinal flora. Through 16S sequencing, fecal non-targeted metabolic sequencing and Spearman correlation analysis, we found that Tongxinluo reversed some of the changes in the intestinal flora and intestinal metabolites caused by HCB and reduced the ratio of *Bacteroides* to Firmicutes to enhance intestinal homeostasis. After intervention with Tongxinluo, the metabolites that contributed to AS were significantly reduced, while the metabolites that played a role in anti-atherosclerosis, such as trans-ferulic acid, DL-3-phenyllactic acid, nitrilotriacetic acid, and hydroxyphenyllactic acid, were significantly increased. Other studies have found that trans-ferulic acid, which is beneficial for improving the stability of vulnerable plaques, can inhibit the inflammatory response of macrophages by inhibiting the NLRP3 pathway. Overall, these results provided new evidence of the interaction among TXL, the intestinal flora, and diseases and indicated that TXL could be used in the clinic to treat anti-atherosclerotic inflammation.

## 2 Materials and Methods

### 2.1 Ethics Statement

The present research program was approved by Ethics Committee of Shandong University Qilu Hospital and conformed to the Helsinki Declaration. All *in vivo* programs comply with the Guidelines for the Care and Use of Laboratory Animals published in the National Institutes of Health (eighth edition, 2011) and the Chinese Ministry of Health Animal Management Code (Item No. 55,2001) (Chen et al., 2018). All procedures were performed in compliance with relevant laws and institutional guidelines.

### 2.2 TXL Preparation

TXL capsule, was sponsored by Shijiazhuang Yiling Pharmaceutical Co., Ltd., includes 12 medicinal components. These materials were originally ground to a fine powder by a micronized and prepared as capsules, which were authenticated and standardized on the basis of marker compounds in the Chinese Pharmacopoeia 2005 (National Pharmacopoeia Committee Pharmacopoeia of China. First Part Chinese Publishing Company of Chemical Industry, Beijing (2015) pp. 1447). Since 2006, with the improvement of micronized technology, the raw materials have been further ground to superfine powder (≤10 μm) and the pharmacodynamic effects of TXL capsule have been doubled. In high-performance liquid chromatography analysis, the similarity of the fingerprints of each batch of TXL was above 95%, indicating that the product quality was stable and controllable (Meng et al., 2014)_._

### 2.3 Animal Model

Sixty-two male New Zealand white rabbits were adaptively fed for a week, and then were randomly divided into control group (n = 12) and experimental group (n = 50). After that, forty-eight experimental rabbits were anesthetized by intravenous injection of sodium pentobarbital (30 mg/kg), and the 4F balloon catheter was inserted into the thoracic aorta through the right femoral artery to cause balloon-induced aortic wall injury. We inflate the balloon under a pressure of 8 atm, and then retract the catheter to the femoral artery. Repeat this process 3 times for each rabbit to ensure that the endothelium of the abdominal aorta is injured. Then these rabbits with balloon-induced aortic endothelium injury were fed with an atherogenic diet containing 1% cholesterol for 8 weeks. Exclude surgical death, the remaining rabbits were randomly divided into four groups (n = 10). One group did not receive medication (HCB group), and the remaining three groups were given high-dose TXL original powder (0.6 g·kg-1·day-1) (Zhang et al., 2009) on the basis of a high-cholesterol diet, for 4 weeks (HCB + TXL4w group), 8 weeks (HCB + TXL8w group), and 12 weeks (HCB + TXL12w group) respectively. At the end of the TXL treatment, the rabbits were pharmacologically triggered as described in previous study (Chen et al., 2007), that is, 0.15 mg/kg of Chinese viper venom (Obtained from the Snake Venom Research Institute of Fujian Medical University) was injected intraperitoneally, and then 30 min later, 0.02 mg/kg histamine (Sigma Chemical Corp., United States). The euthanized rabbits were subjected to pathological studies 24 h after the pharmacological trigger. The body weight of rabbits was monitored throughout the experiment (Chen et al., 2009).

### 2.4 Biochemical Research

Separately, before surgery, drug intervention and pharmacological triggering, blood was drawn from the ear arteries of the rabbits on an empty stomach, and serum was separated by centrifugation at 4°C for 15 min. The concentrations of total cholesterol (TC), triglycerides (TG), high-density lipoprotein cholesterol (HDL-C) and low-density lipoprotein cholesterol (LDL-C) in each sample were measured by enzymatic assays using an automated biochemical analyzer (Roche/Hitachi 917, Block Scientific, Inc., New York, United States). Similarly, the concentrations of IL-1β and tumor necrosis factor alpha (TNF-α) were measured with ELISA kits (enzyme immunoassay).

### 2.5 Histopathological Analysis

#### 2.5.1 Aortic Separation and Histopathology

An overdose of sodium pentobarbital solution was injected intravenously to euthanize the rabbits. The aortas from all of the rabbits were completely separated from the root to the bifurcation of the abdominal aorta. One the part of these aortas fixed in 4% paraformaldehyde for staining with oil red O. And another part, some were cut along the longitudinal axis immediately to observe the plaque rupture and thrombosis, and then, all of the abdominal aortas were preserved. The abdominal aorta was divided into three parts and separately used to make 5 μm thick paraffin sections and frozen sections, or stored in liquid nitrogen. The frozen sections, stained with Sirius Red and Oil-red-O (Santa Cruz Biotechnology, Santa Cruz, CA, United States).

#### 2.5.2 Immunochemistry

The paraffin sections were incubated with primary antibodies against mouse anti-rabbit NLRP3 (Aviva Systems, ARP63297_P050), caspase1 (Aviva Systems, ARP58983_P050), IL- 1β (abcam, ab9722), IL-18(Invitrogen, PA5-79479), MOMA-2 (abcam, ab33451) and α-SMA (CST19245) monoclonal antibody response. The results were analyzed by using a computer-aided morphometric analysis system (Image-Pro Plus 6.0, Media Cybernetics, United States). Measure at least three slices of each sample and take the average. The vulnerability index was calculated as (macrophage staining% + lipid staining%)/(SMC% + collagen fiber%) (Shiomi et al., 2001). Plaque rupture is defined as a plaque with deep injury with a real defect or gap in the fibrous cap that had separated its lipid-rich atheromatous core from the flowing blood, thereby exposing the thrombogenic core of the plaque (Schaar et al., 2004).

### 2.6 Western Blotting Analysis

The abdominal aorta tissue was lysed in RIPA buffer with protease and phosphatase inhibitors, and then the protein extracts were resolved by SDS–PAGE electrophoresis and transferred onto polyvinylidene difluoride (PVDF) membranes (0.45 μm or 0.22 μm; Millipore) (Zhang et al., 2019). Membranes were blocked with 5% skimmed milk for 1 h at room temperature and incubated overnight at 4°C with primary antibodies for NLRP3 (Aviva Systems, ARP63297_P050), caspase1 (Aviva Systems, ARP58983_P050), IL- 1β (abcam, ab9722), IL-18(Invitrogen, PA5-79479)and GAPDH (ZSGB-BIO TA-08). The next day, membranes were incubated with HRP-conjugated secondary antibody. The blots were visualized with chemiluminescence (Millipore) and quantified by densitometry.

### 2.7 Fecal Pellets Collection, DNA Extraction and 16S rRNA Gene Sequencing

#### 2.7.1 Fecal Pellets Collection, DNA Extraction

75% alcohol was used to disinfect the skin around the rabbit’s anus and massage the abdomen to promote defecation. Put fresh fecal pellets immediately into a sterile cryotube and store in liquid nitrogen. Genomic DNA was extracted from the fecal pellets using the SanPrep Column DNA Gel Extraction Kit (Sangon Biotech, B518131) (Costello et al., 2019).

#### 2.7.2 16S rRNA Gene Sequencing

DNA concentration was determined by Nanodrop 2000 (Thermo Scientific) spectrophotometer. The V3-V4 regions of 16S rRNA gene were amplified by PCR using universal primers (Wang et al., 2018a). The sequencing library for these amplicons was constructed according to the manufacturer’s manual. After mixing with PhiX library (40%), the sequencing library was sequenced on MiSeq for 300 bp paired-end reads (Yan et al., 2020).

### 2.8 Bioinformatic Analysis of Sequencing Data

The sequencing data were merged and filtered using fastq software. Then the identification and taxonomic annotation of amplicon sequence variants (ASVs) were performed using Quantitative Insights into Microbial Ecology 2 (QIIME2) software (https://qiime2.org/) and Greengenes 13.8 database according to the manual. The diversity indexes were calculated using QIIME2. The canonical correlation analysis (CCA) and redundancy analysis (RDA) were performed using Calypso online tools (Zheng et al., 2015). The comparison of relative abundances of bacterial taxa of different groups and Spearman correlation analysis were performed using R packages Psych and WilcoxCV tools. The metabolic function of ASVs was predicted using the Tax4Fun package (Aβhauer et al., 2015) with R (v 3.5.0), which converted the SILVA-labeled OTUs into KEGG (Kyoto Encyclopedia of Genes and Genomes) organisms, and then normalized the predictions by the 16 S rRNA copy number (obtained from the NCBI genome annotations). The heatmaps were drawn using R package pheatmap (Yan et al., 2020).

### 2.9 Untargeted Metabolomic Analysis of Fecal Samples

Approximately 100 mg stool was extracted with 1 ml methanol, and 60 μL 2-Chloro-l-phenylalanine and Heptadecanoic acid were added as the internal standard. After vortex, ultrasonic treatment and centrifugation, the supernatant was transferred into a new tube and dried. Then 60 μL 15 mg/ml methoxyamine pyridine and N, O-Bis(trimethylsilyl)trifluoroacetamide reagent were sequentially added and incubated at 37°C. After centrifugation, the supernatant was analyzed using the ACQUITY UPLC system (Waters Corporation, Milford, United States) coupled with AB SCIEX Triple TOF 6600 System (AB SCIEX, Framingham, MA) in positive and negative ion modes according to the manufacturer’s manual (Yan et al., 2020).

### 2.10 Metabolomic Data Analysis

All metabolite ions were normalized and the ions (RSD%<30%) were further analyzed by SIMCA 14.0 (Umetrics, Umeå, Sweden) and XCMS 1.50 software. The orthogonal partial least squares discriminant analysis (OPLS-DA) and its validation were performed after mean centering and unit variance scaling by SIMCA. The differential peak features were selected based on the combination of variable influence on projection (VIP) values (>1) obtained from the OPLS- DA model and *p* values (<0.05) from a Wilcoxon rank-sum test on the normalized peak areas (Langille et al., 2013). Only the peak features with more than 50% non-zero values in at least one group were retained and used to identify the differential metabolites with the reference material database including HMDB, METLIN and that built by Dalian ChemData Solution Information Technology Co., Ltd. The cloud plots were generated using XCMS online (https://xcmsonline.scripps.edu/) (Yan et al., 2020).

### 2.11 Cell Culture

Human acute monocytic leukemia cell line (THP-1) was obtained from the American Type Culture Collection (ATCC) and cultured in RPMI 1640 containing 10% fetal bovine serum (Gibco, United States) and 1% penicillin/streptomycin. Passage of cells within 50 generations. 160nM phorbol myristate acetate (PMA) was used overnight to differentiate cells into macrophages (Chen et al., 2018). THP-1 macrophages were treated with or without trans-ferulic acid (TFA) for 48 h and then stimulated with 200 ng/ml lipopolysaccharide (LPS) for 12 h. TFA was diluted with 1,640 medium stock solution to different concentrations.

### 2.12 Detailed Methods, Data Available and Statistical Analysis

The authors declare that data and methods used to support of the findings presented in this study will be made available from the corresponding author upon reasonable request. Data were presented as mean ± SEM or median (minimum to maximum). The Shapiro-Wilk test was used to evaluate data normality. For normally distributed data, *t*-test and one-way ANOV A test with post hoc test (Turky or Dunnett) for multiple testing correction were used for comparison between two groups and multi-group comparison, respectively. For data not distributed normally, Wilcoxon rank sum test and Kruskal–Wallis test with post hoc test (Dunn) for multiple testing correction were used for comparison between two groups and multi-group comparison, respectively. For certain multiple comparison or statistical analysis of omic data including metabolomic and 16S rRNA gene sequencing data, *t*-test or Wilcoxon rank sum test adjusted by Benjamini–Hochberg method with FDR<5% was used. Statistical analysis was performed using GraphPad Prism v8 and R packages. The *p* < 0.05 or adjusted *p* value < 0.05 was considered statistically significant. All experiments were repeated independently at least thrice.

## 3 Results

### 3.1 HCB Significantly Promoted the Formation of Plaques and Enhanced Inflammation in Rabbits

**FIGURE 1:**
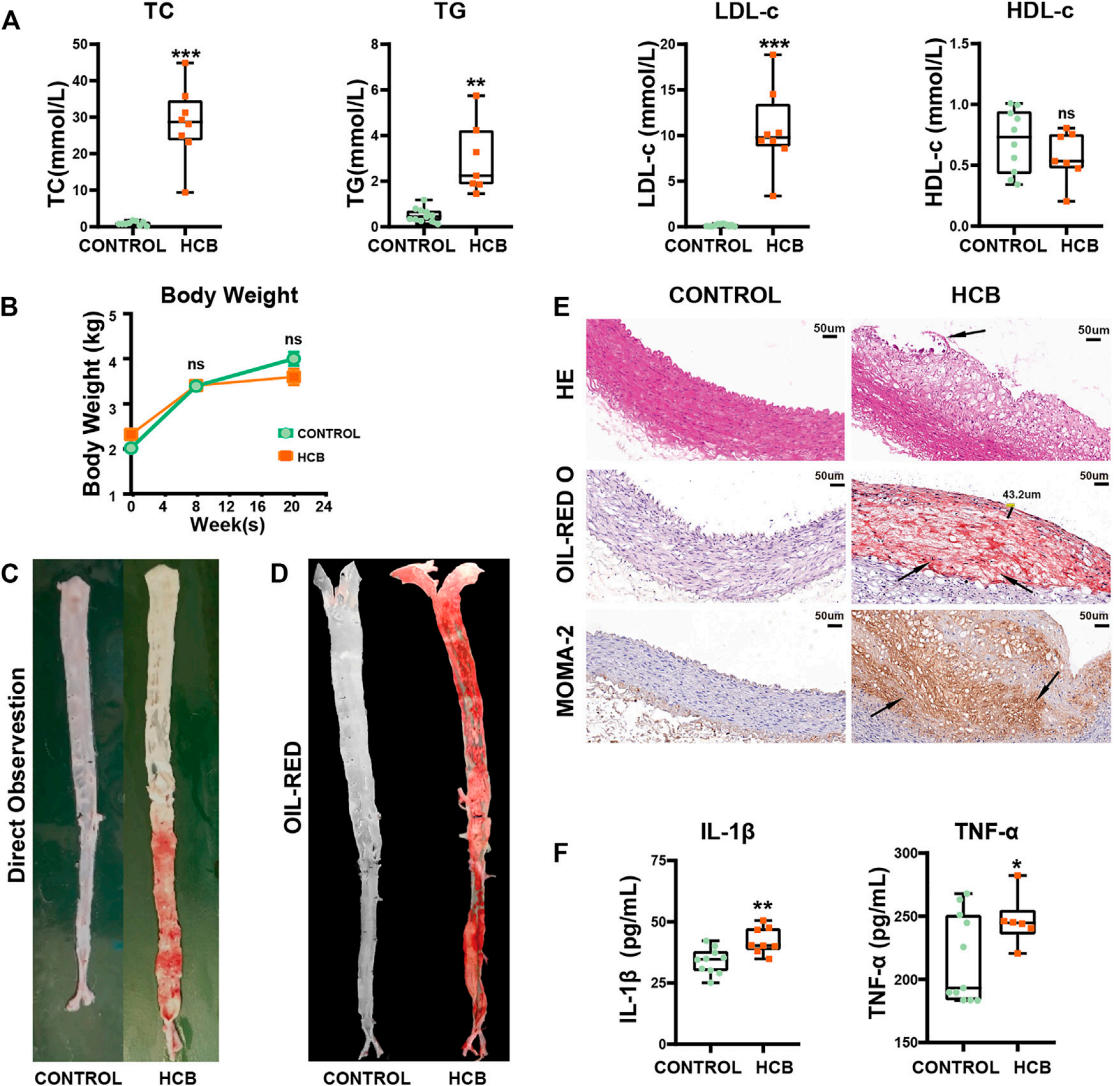
HCB significantly promoted the formation of plaques and enhanced the level of inflammation. CONTROL: normal diet (n = 12); HCB: high cholesterol diet and balloon injury (n = 8); w: weeks **(A)** The changes of Blood lipids of New Zealand White Rabbits in HCB and CONTROL groups measured by enzymatic determination. **(B)** The changes in body weight of two groups **(C,D)** the formation of plaques in two groups. The general view directly **(C)** and Oil Red O staining of the entire aorta (D) in the CONTROL group (left) and HCB group (right). Oil red O (ORO) staining was applied to show lipid **(E)**= HE staining, Oil Red O staining and MOMA-2 immunohistochemical staining of abdominal aortic cross-sections. The black line segment marked 43.2 μm represents the measured thickness of the fiber cap was 43.2 μm. The area pointed by the black arrows represent ruptured plaques, lipid cores, and tan-stained macrophages. **(F)** The changes of IL-1β and TNF-α of New Zealand White Rabbit in two groups measured by ELISA Kits. Data presented as median (minimum to maximum) (A, F), and the mean ± SEM (B). Non-parametric test was used for statistical tests in A, B and F. *: *p* < 0.05; **: *p* < 0.01, ***: *p* < 0.001.

Compared with those in the control group, which was fed a normal diet, the levels of triglycerides (TG), total cholesterol (TC) and low-density lipoprotein cholesterol (LDL-c) in serum in the HCB group were significantly increased, and high-density lipoprotein (HDL-c) was not significantly change (Figure 1A). Additionally, there was no significant difference in the body weight of the two groups (Figure 1B). To assess the formation of plaques, we separated the thoracic and abdominal aortas and then observed plaque rupture and thrombosis via a longitudinal incision. The intima of the vascular wall in the control group was smooth without plaques, but in the HCB group, both the thoracic aorta and abdominal aortas had obvious bulging white plaques, and the inner wall of the abdominal aorta showed obvious damage, and its color was sanguine (Figure 1C). Oil red O staining of the entire aorta (Figure 1D; Supplementary Figure S1A) also showed large areas of plaque formation. HE staining, Oil red O staining and macrophage (marked with MOMA-2) staining of abdominal aorta sections also showed obvious plaques in the HCB group that were characterized by thin (thickness <65 µm) or cracked fibrous caps, a large lipid core and a large number of macrophages (Figure 1E), which were consistent with the features of vulnerable plaques. To identify inflammation in the HCB and control groups, we used ELISA to detect the levels of IL-1β and TNF-α in the serum of rabbits and found that the levels of these two factors in the HCB group were significantly higher than those in the control group. In summary, the above experiments confirmed that the high cholesterol diet and balloon injury promoted the formation of vulnerable plaques and enhanced the inflammatory response in rabbits.

### 3.2 TXL Improved the Stability of Plaques and Alleviated Inflammation Induced by HCB in Rabbits in a Non-lipid-dependent Manner

**FIGURE 2:**
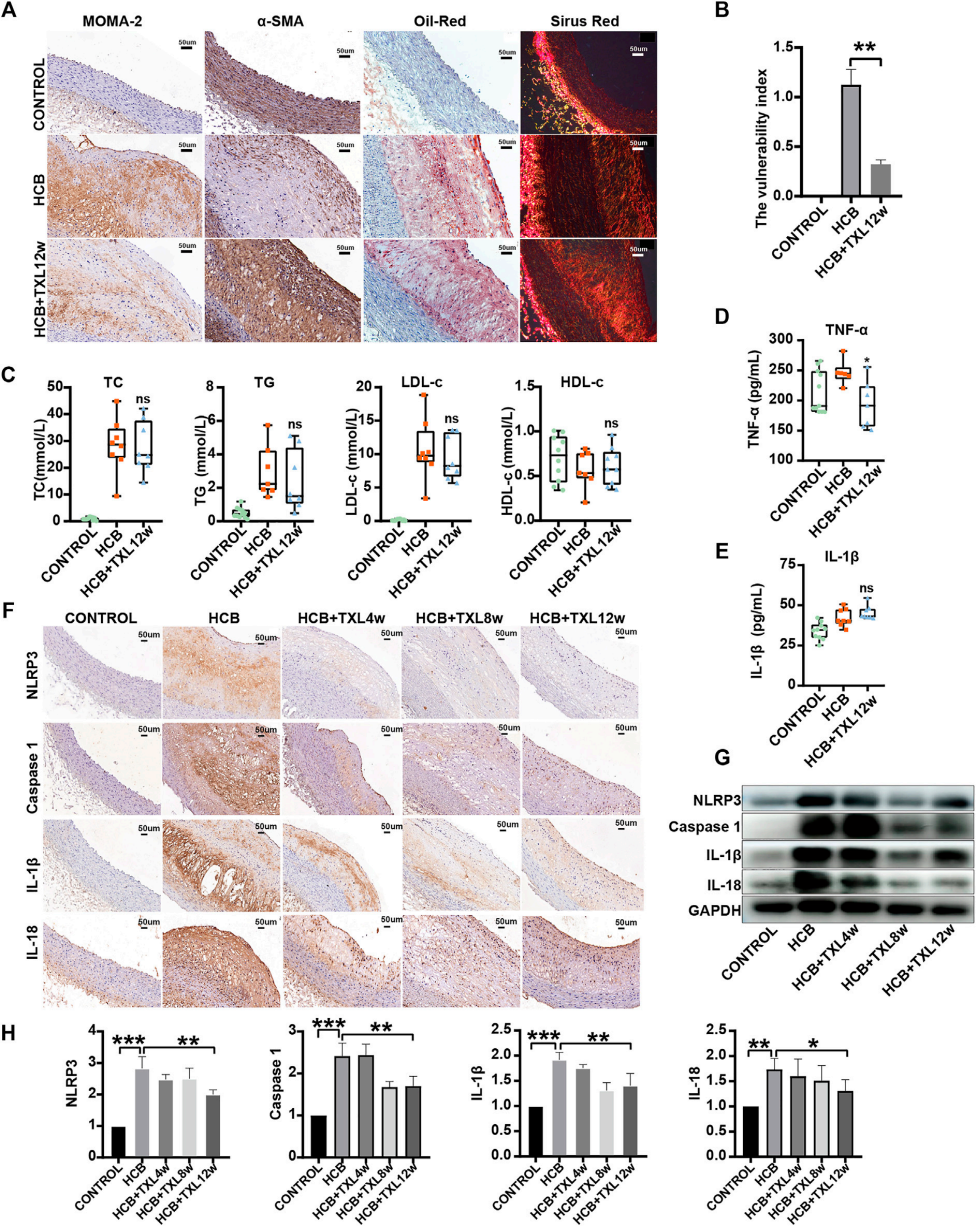
Tongxinluo improved the stability of HCB-induced plaques and alleviated inflammation through non-lipid-dependent pathways in rabbits. CONTROL: normal diet (n = 12); HCB: high cholesterol diet and balloon injury (n = 8); HCB + TXL 4w: HCB and treated with TXL for 4 weeks (n = 7). HCB + TXL 8w: HCB and treated with TXL for 8 weeks (n = 8). HCB + TXL 12w: HCB and treated with TXL for 12 weeks (n = 10) **(A)** Plaque’s stability test results. Oil red O staining was applied to show lipid in abdominal aorta. Sirius red staining was used to detect collagen fibers. Abdominal aorta sections were stained for macrophages (MOMA-2) or vascular smooth muscle cells (α-SMA). bar = 50 μm **(B)** Plaque vulnerability index statistics. Data are shown as the mean ± S.E.M. **: *p* < 0.01. **(C,D,E)** The changes of Blood lipids (C), Serum inflammatory factors TNF-α (D) andIL-1β **(E)** of New Zealand White Rabbit in normal diet (CONTROL, n = 12), high cholesterol diet (HCB, n = 8) and high cholesterol diet with TXL treatment for 12 weeks (HCB + TXL 12w, n = 10), measured by enzymatic determination. Non-parametric test was used for statistical tests. Data were presented as median (minimum to maximum) **(F)** Histopathological results. Immunohistological staining of the abdominal aortic cross-section showing dense, positive NLRP3/Caspase-1/IL-1β/IL-18 in the five different treatment groups. **(G,H)** Western blotting to quantitate NLRP3/Caspase-1/IL-1β/IL-18 protein expression **(G)**, and non-parametric test was used for statistical, data were shown as the mean ± S. E. M **(H)**. *: *p* < 0.05; **: *p* < 0.01, ***: *p* < 0.001.

As mentioned above, studies have shown that TXL intervention may reduce the plaque area and improve atherosclerosis, but the specific mechanisms by which these effects occur are not clear. To further clarify the role and mechanism of TXL in atherosclerotic vulnerable plaques, we collected the abdominal aorta and peripheral serum of rabbits and then detected the stability of plaques and inflammatory factors. We produced abdominal aorta slices for staining to detect the proportions of lipids, collagen, macrophages (MOMA-2) and smooth muscle cells (SMCs) in plaques and calculated the vulnerability index: (macrophage% + lipid %)/(SMC% + collagen fibers%). The results showed that compared with that of the plaques of the abdominal aorta in rabbits in the HCB group, the plaques of the abdominal aorta in rabbits treated with TXL for 12 weeks had a significantly lower vulnerability index (Figure 2A,B). We measured blood lipids by an enzymatic method and found that TXL intervention did not reduce the concentrations of TG, TC, or LDL-c and had no significant effect on HDL-c (Figure 2C, Supplementary Table S3). Clearly, lowering blood lipids is not the mechanism by which TXL improves atherosclerosis. Furthermore, we used ELISA to detect serum inflammatory factors and showed that TXL intervention significantly reduced the concentration of TNF-α (Figure 2D, Supplementary Table S3) but not the concentration of IL-1β (Figure 2E, Supplementary Table S3). Therefore, due to its effect on reducing the concentration of TNF-α, we believe that TXL may alleviate the body’s inflammatory response. To further explore the anti-inflammatory pathway of TXL, we determined the expression of members of the NLRP3 pathway, which plays a pivotal role in the inflammatory response. Western blot analysis (Figure 2G,H) and immunohistochemistry (Figure 2F) showed that in vascular tissue, HCB significantly increased the expression of NLRP3, caspase-1, IL-1β and IL-18, but after TXL intervention, the expression of these factors was significantly reduced. Therefore, TXL can exert its anti-inflammatory effects by inhibiting the expression of members of the NLRP3 inflammatory pathway.

### 3.3 TXL Significantly Changed the Structure and Composition of the Intestinal Flora of Rabbits

**FIGURE 3:**
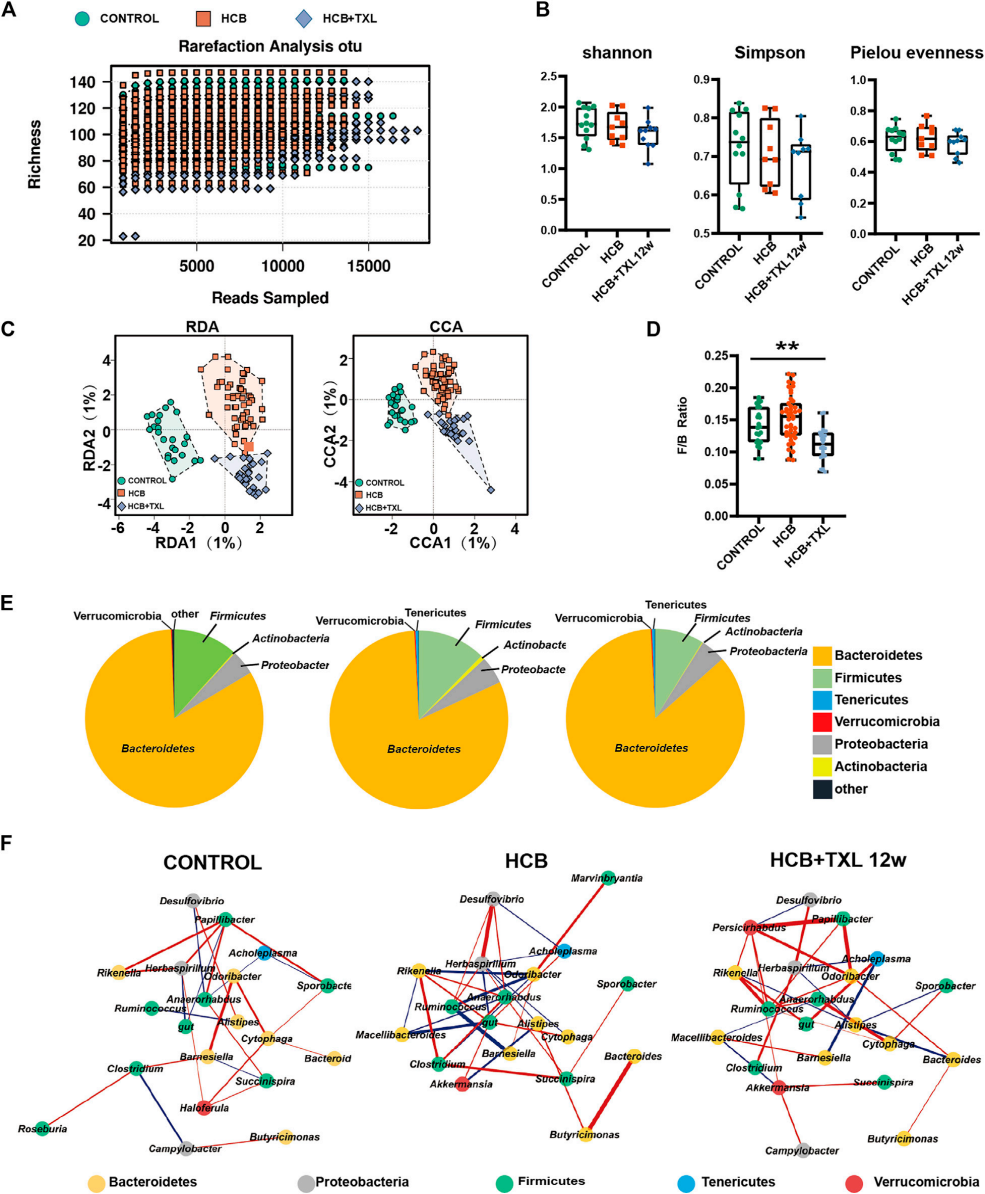
HCB and TXL significantly changed intestinal flora composition **(A)** Rarefaction curves of ASV numbers detected by 200 random samplings in CONTROL (n = 24), HCB (n = 52) and TXL treatment (n = 28) groups. The slope of the curve for each sample is close to zero when the sequencing depth is sufficient to cover most of the intestinal flora **(B)** Analysis of α diversity of intestinal flora in rabbits. Shannon index, Simpson index and Pielou evenness were performed. **(C)** Analysis of β diversity of intestinal flora in rabbits. RDA and CCA were performed to calculate the distances between fecal samples from the rabbits of CONTROL, HCB and TXL + TXL12w groups. Each point represents a sample. A clear separation is observed between the samples of different groups **(D)** The ratio of Firmicutes to Bacteroidetes (F/B ratio) in CONTROL (n = 24), HCB (n = 52) and TXL treatment (n = 28) groups. Data were presented as median (minimum to maximum). Wilcoxon rank-sum test was used for statistical tests. **: *p* < 0.01 **(E)** The pie chart of the six most abundant bacterial phyla in CONTROL (n = 24), HCB (n = 52) and TXL treatment (n = 28) groups **(F)** Correlation network of gut microbiota in CONTROL (n = 24), HCB (n = 52) and TXL treatment (n = 28) groups. The significant strong Spearman correlations (|r|>0.5 and FDR-adjusted *p* value < 0.05, FDR<5%) among the intestinal bacterial genera are presented in the networks. The red and blue edges show positive and negative correlations, respectively. The spot colors represent different bacterial phyla. The thickness of the lines denotes correlation strength.

In recent years, an increasing number of studies have shown that the efficacy of Chinese medicine is inextricably linked to the effects of intestinal microbes, and imbalances of intestinal microbes are closely related to inflammation and many chronic diseases. To determine whether the anti-inflammatory effect of TXL was related to intestinal microbes, we collected rabbit feces from each group and performed 16S rRNA gene sequencing. The sequencing depth was 1–1.5 million reads/sample, which met the analysis requirements (Figure 3A). Among the three groups (control, HCB and HCB + TXL 12 w), the Shannon index, Simpson index and Pielou’s evenness index did not show significant differences (Figure 3B), indicating that HCB and TXL intervention had no significant effect on the diversity of the intestinal flora. However, both CCA and RDA, based on amplicon sequence variations, indicated that the three groups were significantly separated (Figure 3C), and the trend of the separation indicated that TXL could obviously reverse the changes induced by HCB.

We determined the relative abundance of the phyla and found that compared with those in the control group, intestinal *Bacteroides* (B) was significantly reduced in the HCB group (Supplementary Figure S1B) and the Firmicutes (F)/Bacteroidetes (F/B) ratio was significantly increased (Figure 3D). These results are consistent with previous research results. However, after TXL intervention, the abundance of *Bacteroides* increased significantly (Supplementary Figure S1B) and the F/B ratio decreased (Figure 3D), suggesting that HCB could induce an imbalance of the intestinal flora and TXL intervention could alleviate this imbalance. In addition, HCB induced decreases in the abundances of Firmicutes and Actinomycetes (A), and TXL reversed these changes (Figure 3E, Supplementary Figure S1C,D). To clarify the influence of HCB and TXL on interactions between bacteria, we performed Spearman correlation analysis on bacteria at the genus level and used Cytoscape to construct a correlation network. The network diagram (Figure 3F) showed that HCB significantly increased the negative correlation between many intestinal bacteria, especially *Bacteroides* and Firmicutes, such as *Ruminococcus* (F) and *Barnesiella* (B), *Ruminococcus* (F) and *Odoribacter* (B), *Macellibacteroides* (B) and *gut* (F), and *Macellibacteroides* (B) and *Anaerohabdus* (F). These results indicated that HCB disrupted the symbiotic relationship between Firmicutes and *Bacteroides* and cause their normal ratio to be out of balance. However, after intervention with Tongxinluo, these negative correlations weakened or even disappeared. In addition, in the control group, *Papillibacter* (F) was positively correlated with *Rikenella* (B), *Herbaspirillum* (P), *Anaerorhabdus* (F), and *Sporobacter*, but HCB disrupted these positive correlations, while intervention with TXL caused the correlations to approximately return to the levels found in the control group.

**FIGURE 4:**
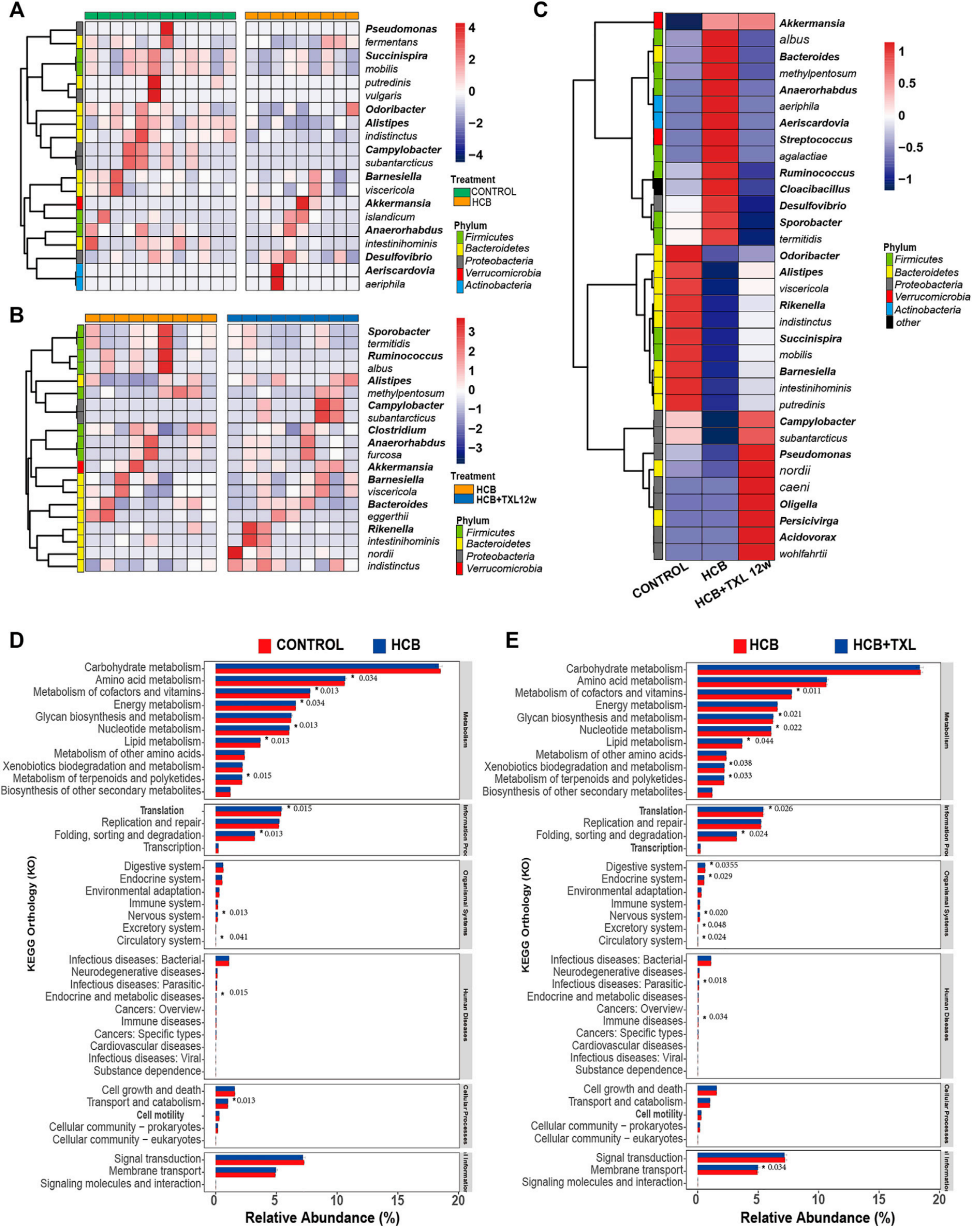
TXL changes the relative abundances of intestinal bacteria **(A)** Heatmap of the relative abundances of the 20 most abundant intestinal bacteria which significantly changed in HCB (n = 9) compared with those in CONTROL (n = 12) group (FDR-adjusted *p* < 0.05, FDR<5%) **(B)** Heatmap of the relative abundances of the 20 most abundant intestinal bacteria which were significantly changed in HCB + TXL 12w treatment (n = 12) compared with those in HCB (n = 12) group (FDR-adjusted *p* < 0.05, FDR<5%) **(C)** Heatmap of the relative abundances of the 33 most abundant intestinal bacteria which were significantly changed in CONTROL (n = 12), HCB (n = 9) and HCB + TXL 12w treatment (n = 10) groups of the rabbits (FDR-adjusted *p* < 0.05, FDR<5%). The color bar indicates Z score that represents the relative abundance. Z score<0 and>0 means the relative abundance is lower and higher than the mean. Row names thickened represent bacteria genus, otherwise represent bacteria species. **(D,E)** Tax4Fun predicts the function of microorganisms in the sample. *: *p* < 0.05, the number after the “*” are *p* value.

To clarify specific differences in bacteria, we performed Wilcoxon difference analysis on the 16S results of the three groups at the genus and species levels. The results showed (Figure 4A) that compared with those in control group, the bacterial relative abundance of 20 species or genera in the HCB group changed significantly. For example, *Succinispira mobilis*, *Alipipes*, *indistinctus*, *Campylobacter*, *Odoribacter*, *subantarcticus* and other bacteria were significantly reduced, while *Akkermansia*, *islandicum*, *Desulfovibrio*, and other bacteria were increased. After TXL intervention, the relative abundance of some fecal flora also changed significantly, including *Alipipes*, *Campylobacter*, *Rikenella*, *indistinctus*, *viscericola*, *nordii*, *subantarcticus* and others, which were increased, and *Ruminococcus* and *albus*, which were decreased (Figure 4B). Among these different bacteria, TXL significantly reversed the relative abundance changes of 33 intestinal genera or species (Figure 4C), of which 13 were significantly reduced, seven Firmicutes, 1 Bacteroidetes, 1 Proteobacteria, 1 Verrucomicrobia, two Actinobacteria, and 1 Synergistetes, 20 were significantly increased, 10 Bacteroidetes, seven Proteobacteria, two Firmicutes, and 1 Verrucomicrobia.

Next, we used Tax4fun to predict the function of the flora in the samples. The results showed that although the magnitude of the differences among the groups was relatively small, there were still some significant differences. Compared with those in the control group, the HCB group had significant changes in amino acid metabolism, cofactor and vitamin metabolism, energy metabolism, nucleotide metabolism, lipid metabolism, and terpenoid and polyketide metabolism (Figure 4D). Compared with that in the HCB group, metabolism in the TXL group also changed significantly, such as cofactor and vitamin metabolism, glycan biosynthesis and metabolism, nucleotide metabolism, lipid metabolism, xenobiotic biodegradation and metabolism, and terpenoid and polyketide metabolism. (Figure 4E). The above results indicated that the changes in the intestinal flora caused by HCB and TXL intervention led to significant changes in the metabolism of the intestinal flora.

### 3.4 The Metabolic Characteristics of the Fecal Flora Were Significantly Changed by HCB and TXL Intervention

**FIGURE 5:**
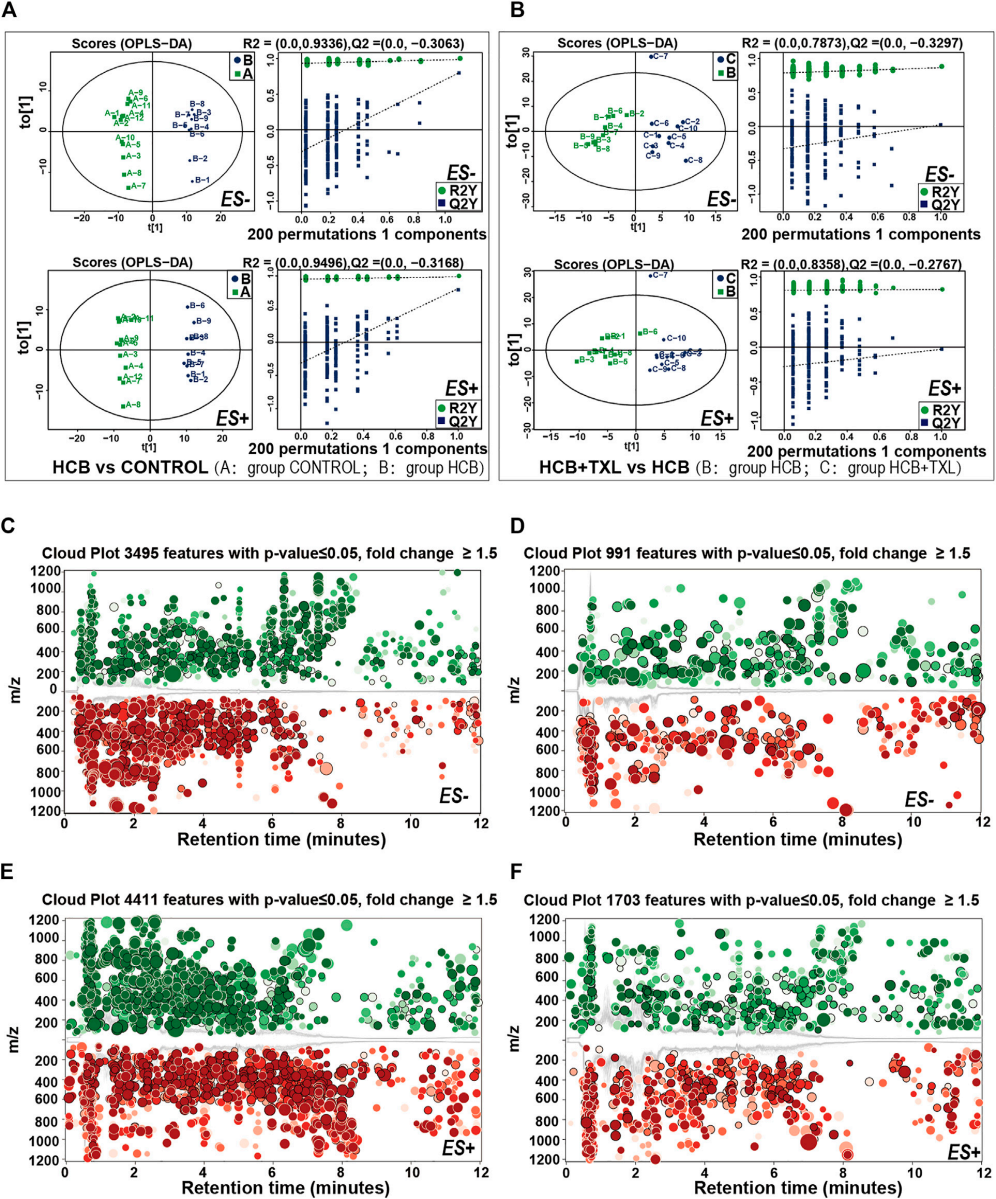
High-performance liquid chromatography-mass spectrometry explored the intestinal metabolic profiling **(A,B)** The plots of OPLS-DA scores of all peak features in positive (ES+) and negative (ES-) ion modes from the untargeted metabolomics analysis of stool samples of the rabbits in CONTROL (n = 12), HCB (n = 9) and HCB + TXL 12w groups. Validation of the OPLS-DA model via permutation test (times = 200). R2 measures the goodness of fit and Q2 measures the predictive ability of the model. The criterion for model validity is that the regression line of the Q2-points (blue dotted line) intersects the vertical solid line (on the left) below zero **(C,E)** Cloud plots of the peak features of intestinal metabolites which significantly changed in HCB group (n = 9) compared with those of CONTROL (n = 12) group in negative ion mode (ES-) (C) and positive ion mode (ES+) **(E)** **(D,F)** Cloud plots of the peak features of intestinal metabolites which significantly changed in HCB + TXL 12w group (n = 10) compared with those of HCB group in negative ion mode (ES-) **(D)** and positive ion mode (ES+) **(F)**. Red and Green circles indicate the significantly increased and decreased metabolites, respectively, (fold change>1.5, *p* < 0.05). The color tone indicates *p*-value: a dark color indicates a small *p* value. The circle radius indicates the fold change of corresponding peak features.

As an important digestive and metabolic organ, the intestine can degrade or transform a variety of substances through the action of microorganisms, and then, these metabolites can affect the host’s physiology. Based on the prediction of the fecal flora function among the different groups, we know that changes in the composition of the fecal flora can lead to significant changes in metabolic pathways. To confirm this prediction and to clarify the specific changes in metabolites and metabolic pathways, we collected faeces from rabbits and used high-performance liquid chromatography-mass spectrometry technology to perform non-targeted metabolomics analysis. Comparing the HCB group and the control group in the positive ion (ES+) (Figure 5E) and negative ion (ES-) (Figure 5C) modes, a total of 4,411 and 3,495 peak features with significant changes were identified, respectively. We used orthogonal partial least squares discriminant analysis (OPLS-DA) to cluster these peaks and performed 200 permutation tests to obtain verification maps. The results showed that the metabolic clusters of the control and HCB groups were separated from each other (Figure 5A). These results indicated that HCB caused significant changes of intestinal metabolites. Similarly, when we compared the HCB + TXL12w group with the HCB group, 1703 and 991 peak characteristics were identified to be changed significantly in the positive ion (ES+) (Figure 5F) and negative ion (ES-) (Figure 5D) modes, respectively. Additionally, OPLS-DA and 200 permutation tests were performed to cluster the peaks and obtain verification maps, and the results showed that the metabolic clusters of the HCB and HCB + TXL 12 w groups were separated from each other (Figure 5B).

**FIGURE 6:**
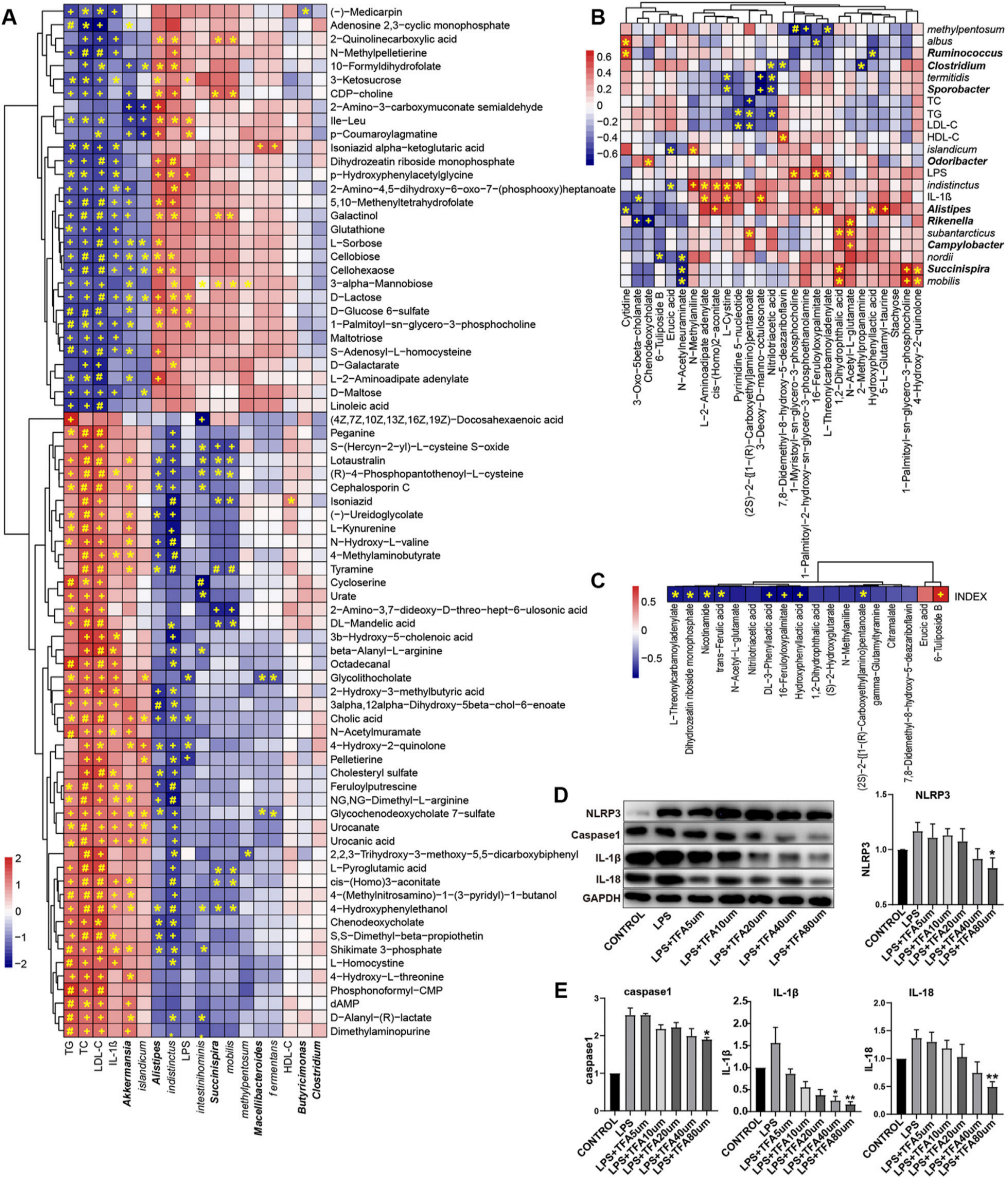
The significant changes in metabolic profiles of gut microbiota in high cholesterol diet and TXL treatment rabbits **(A)** Heatmap of Spearman correlations between the most abundant bacteria whose abundances significantly changed and serum factors in HCB rabbits and the most abundant metabolites with important functions and significant differences. n = 9. **(B)** Heatmap of Spearman correlations between the most abundant bacteria whose abundances significantly changed and serum factors in HCB + TXL 12w rabbits and the most abundant metabolites with important functions and significant differences. n = 10 **(C)** Heatmap of Spearman correlations between the plaque vulnerability index and the most abundant metabolites with important functions and significant differences. n = 10.The color bar with numbers indicates the correlation coefficients. Row names thickened represent bacteria genus, otherwise represent bacteria species. r: the spearman correlation coefficient; p: statistical significance. Adj: adjusted. T-test adjusted by Benjamini Hochberg procedure to control FDR<5% (A, B, C) were used for statistical tests. At FDR<5%, *: FDR-adjusted *p* < 0.05; +: FDR-adjusted *p* < 0.01; #: FDR-adjusted *p* < 0.001. **(D,E)** Western blotting to quantitate NLRP3/Caspase-1/IL-1β/IL-18 protein expression, all groups were stimulated by LPS except the CONTROL group, and non-parametric test was used for statistical, data were shown as the mean ± S. E. M. The horizontal note number represents the concentration of trans-ferulic acid. *: *p* < 0.05; **: *p* < 0.01.

Then, we used tandem mass spectrometry to analyze the characteristics of the significantly changed peaks and identified metabolites by combining their precise molecular weight and structural information from the compound structure database. We found that these metabolites included amino acid derivatives, bile acids, carbohydrates, lipids, fatty acids, phenols, etc (Supplementary Table S1), many of which are directly produced or regulated by intestinal bacteria. To explore the functional significance of these metabolic perturbations in HCB rabbits, 77 annotated metabolites with the most significant differences were selected, and the Spearman correlation coefficients between them and vascular inflammatory factors, cholesterol and different bacteria were calculated. Through this analysis, we found obvious correlations between metabolites and a variety of bacteria and factors (|r|>0.5, *p* < 0.05 after Roosevelt adjustment) (Figure 6A). For example, significantly positive correlations were found between dopamine, cholic acid, lithocholic acid, tyramine, and l-pyroglutamic acid and TC and LDL-c, while Ile-Leu, maltotriose, and 2-oxoadipate were significantly negatively correlated with TC and LDL-c. According to previous studies, these metabolites are inextricably linked with AS-related diseases. In addition, among the metabolites, glycocholate, 3β-hydroxy-5-cholenoic acid, xylitol, and urocanic acid were significantly positively correlated with IL-1β, while d-maltose, daidzein, folate, maltotriose, d-lactose, l-sucrose, and others were significantly negatively correlated with IL-1β. Some differential bacteria, such as *Akkermansia*, *islandicum*, *Alistipes*, *indistinctus*, *intestinihominis*, and *Succinispira mobilis*, were also significantly correlated with many metabolites, as shown in Figure 6.

Compared with those in the HCB group, the fecal metabolites erucic acid, N-acetylneuraminate, chenodeoxycholate, and 6-tuliposide B in the TXL intervention group (Figure 6B, Supplementary Table S2) were significantly reduced, while DL-3-phenyllactic acid, 1-palmitoyl-2-hydroxy-Sn-glycero-3-phosphoethanolamine, nitrilotriacetic acid, hydroxyphenyllactic acid, trans-ferulic acid (TFA) and others were significantly increased. Spearman correlation analysis of the metabolites, the fecal flora and related factors showed that erucic acid was significantly negatively correlated with *indistinctus* and *islandicum* and that 6-tuliposide B was significantly negatively correlated with *nordii*. In the TXL intervention group, the significantly increased metabolites, such as L-2-aminoadipate adenylate, cis-(Homo)2-aconitate, and pyrimidine 5′-nucleotide, were also significantly negatively correlated with *indistinctus*. Spearman correlation analysis of the differential metabolites and the plaque vulnerability index (Figure 6C) showed that DL-3-phenyllactic acid, nitrilotriacetic acid, hydroxyphenyllactic acid, TFA, and others were significantly negatively correlated with the vulnerability index, but 6-Tuliposide B was significantly positively correlated with the vulnerability index. Among these metabolites, TFA and nicotinamide have been proven to improve inflammation (Perez-Ternero et al., 2017; Elhassan et al., 2019), and a previous study showed that hydroxyphenyllactic acid could inhibit the expression of IL-1β(Bürgel et al., 2020).

The above results indicate that HCB caused significant changes in the intestinal flora and flora-related metabolites. Additionally, the changed metabolites were closely related to the key factors of atherosclerosis, which provides new evidence regarding the mechanisms of atherosclerosis and vulnerable plaques. The changes in the intestinal flora and its related metabolites caused by TXL intervention also provide new evidence regarding the mechanisms by which TXL improves the stability of atherosclerotic plaques.

### 3.5 TFA Inhibited the NLRP3 Inflammatory Pathway in THP1-Induced Macrophages

Spearman correlation analysis showed that DL-3-phenyllactic acid, nitrilotriacetic acid, hydroxyphenyllactic acid, TFA and other metabolites were significantly negatively correlated with the vulnerability index, indicating that they might play an important role in stabling vulnerable plaques. In particular, TFA has been proven to have a potentially beneficial effect on health in previous studies. Therefore, we further verified whether TFA could be used on stabling vulnerable plaques through inflammatory pathways. We used LPS to induce THP1 cells to differentiate into macrophages and then administered TFA via concentration gradients and time gradients. Western blot analysis was used to determine the effect of TFA on the NLRP3 pathway. The results showed that TFA inhibited the expression of NLRP3, caspase-1, IL-1β and IL-18 in a concentration-dependent manner (Figures 6D,E), and the higher the TFA concentration, the more obvious the inhibitory effect. In our study, we observed that when the concentration of TFA is 80um, a significant inhibitory effect was exhibited.

## 4 Discussion

In this study, we generated a high-cholesterol diet and balloon injury (HCB)-induced atherosclerotic vulnerable plaque model. Then, we confirmed that HCB could increase the concentration of blood lipids and serum inflammatory factors in rabbits and promote the expression the NLRP3 inflammasome and its downstream factors in abdominal aorta plaques. Additionally, we found that intervention with the drug TXL could significantly improve the stability of atherosclerotic plaques and inhibit the expression of the NLRP3 inflammasome and its downstream factors in abdominal aorta plaques. At the same time, we found that TXL significantly changed the structure and function of the intestinal flora of HCB rabbits, reversed the imbalance of the intestinal flora, and significantly increased the abundance of trans-ferulic acid, a metabolite related to the intestinal flora. Trans-ferulic acid could inhibit the expression of members of the NLRP3 inflammatory pathway in macrophages, which was beneficial for improving the stability of plaques.

There is now a large amount of experimental and clinical evidence indicating that atherosclerosis is a chronic inflammatory disease, and inflammation is caused by the occurrence and development of atherosclerosis (Wolf and Ley, 2019). Similarly, inflammation is one of the most important characteristics of vulnerable plaques (Janoudi et al., 2016). Macrophages, as the key cells in the inflammatory response, are also involved in the whole process, from the formation of lipid streaks to the rupture of plaques. During this process, NLRP3 inflammasome and pro-IL-β are overexpressed (Galis et al., 1994; Pasini et al., 2007). Some clinical studies, such as CANTOS, have shown that inhibiting the expression of IL-1β is crucial for reducing cardiovascular events. Some scholars have also proposed that inhibition of inflammation by antagonists of IL-1β or agents that dissolve or prevent cholesterol crystal formation may stabilize vulnerable plaques (Janoudi et al., 2016). Therefore, inhibiting the expression of the NLRP3 inflammasome and related inflammatory factors in vascular plaques and macrophages is essential to stable vulnerable plaques. In our study, we confirmed that TXL could significantly reduce the concentration of TNF-α in serum, suggesting that TXL could improve the inflammatory state of the body. Moreover, TXL could inhibit the expression of the NLRP3 inflammasome and its downstream factors caspase-1, IL-1β and IL-18 in vascular tissue, and trans-ferulic acid, which is a metabolite of TXL created by the intestinal flora, could also inhibit the expression of members of the NLRP3 pathway in macrophages. These results provide new evidence for the clinical application of TXL to improve the inflammatory state of patients with atherosclerotic diseases and stabilize vulnerable plaques in patients. Although Tongxinluo only inhibits the expression of IL-1β in vascular plaques and does not significantly reduce its concentration in serum, in patients with atherosclerosis, IL-1β is mainly located in the vascular plaques (Galea et al., 1996), so, it can also be partially proved that Tongxinluo plays an important role in inhibiting atherosclerotic inflammation. Our immunohistochemical staining results also confirmed that TXL intervention could significantly inhibit the expression of IL-1β in plaques (Figure 2F). The above results all indicate that Tongxinluo can inhibit the expression of the NLRP3 inflammasome and its downstream factor IL-1β in blood vessels and has obvious anti-inflammatory effects.

In recent years, the intestinal flora and its metabolites have been considered to play important roles in the regulation of inflammation and atherosclerosis. Such as *Alitipes* (Shao et al., 2018; Thingholm et al., 2019; Wu et al., 2020), *Faecalibacterium* (Yin et al., 2015; van den Munckhof et al., 2018), and *Bacteroides* (Matziouridou et al., 2016; Liu et al., 2020), are significantly reduced in atherosclerosis and inflammation-related diseases, some drugs with anti-atherosclerotic effects, such as berberine, can restore the abundance of *Alistipes* in the intestinal flora. *Faecalibacterium. Prausnitzii* can produce rich butyrate (Flint et al., 2012; Miquel et al., 2013), which will prevents atherosclerosis (Bultman, 2018). In our study, 16S rRNA gene sequencing analysis also found that in the HCB-induced atherosclerosis-vulnerable plaque rabbit model, the abundances of *Alipipes*, *Campylobacter*, *Odoribacter*, *indistinctus*, *Putredinis* and some other species of bacteria were significantly reduced, which is consistent with previous research results. However, for some bacteria, such as *Akkermansia*, the changes in their abundance are different from those found in previous studies. These different results deserve our careful consideration. However, inconsistencies of the significantly changed gut bacteria and metabolites were present not only in our study but also in many previous studies. There are many reasons for these inconsistent results. For example, the different experimental models can cause differences in results. There is a great difference between the vulnerable plaque rabbit model and the atherosclerotic mouse model. First, the animals are different; C57BL/6J mice, ApoE^−/-^ mice in a C57BL/6J background and rabbits have great difference in their genetic backgrounds. Second, the diet and living conditions of animals are different. In addition, the software and supporting databases used are different, such as Usearch and ribosome database projects, QIIME2 and GreenGenes reference databases. There are also differences in the mass spectrometry parameters and sensitivity, such as the Pegasus IV mass spectrometer, Maui-SILVIA software, the AB SCIEX 6600 mass spectrometer, XCMS software and Metaboanalyst, which can also cause differences in results. Therefore, the changing trend of bacteria should be considered according to the experimental background used, and more similar studies are needed to verify these findings.

As one of the important components of medicine, traditional Chinese medicine (TCM) has been used in China and its surrounding areas for thousands of years. Its rich history of being used to treat diseases and the continuous development of drugs have also gradually popularized Chinese medicine worldwide (Wang et al., 2018b). For example, the role of artemisinin in the treatment of malaria has been accepted worldwide (Tu, 2011). Jinhua Qinggan granules, Lianhua Qingwen capsule, and Xuebijing injection have also been shown to play important roles in the treatment of coronavirus disease 2019 (COVID-19) (Huang et al., 2020). However, the complex theories of TCM and the unclear biological activity of compounds still largely limit the rational use of TCM in clinical practice and its acceptance by many Western populations (Feng et al., 2019). In fact, many Chinese herbal medicines have similar activities to those of small chemical molecules, the effects of which are caused by the intestinal flora. However, previous research on Chinese herbal medicines has neglected the important effects of the intestinal flora. Only in the past 10 years, has research on the relationships between Chinese medicine and the intestinal flora begun to receive attention. In fact, there are complex interactions between the intestinal flora and Chinese herbal medicine, including (Herrington et al., 2016) biological conversion of the compounds in Chinese herbal medicines into metabolites, which have different bioavailabilities and bioactivities/toxicities that their precursors, by the intestinal flora; (Libby et al., 2011); regulation of the composition of the intestinal flora by Chinese herbal medicines, which ameliorates dysfunctions of the intestinal flora as well as associated pathological conditions; and (Xu et al., 2019) mediation of the interactions (synergistic and antagonistic) among the multiple compounds in Chinese herbal medicines by the intestinal flora. There are many kinds of intestinal flora in our intestines, which transform Chinese herbal compounds into a variety of metabolic components and then modify them into active components. We call these active components postbiotics, which are chemicals that will play real therapeutic roles in the future (Lin et al., 2020). For example, a study on a polysaccharide from *Cordyceps sinensis* showed that its effect was not directly produced by Cordyceps, but a kind of bacteria induced by Cordyceps called *Parabacteroides goldsteinii*, whose effect can be independent of Cordyceps sinensis but similarly to it. *Dendrobium* can reduce blood sugar, body weight, and the low-density lipoprotein cholesterol (LDL-C) level and increase the insulin (INS) level in mice. After Dendrobium treatment, the balance of the intestinal flora in mice was improved, the *Bacteroidetes* to *Firmicutes* ratio and the relative abundance of *Prevotella/Akkermansia* were increased, and the relative abundance of *S24-7/Rikenella/Escherichia coli* was reduced (Li et al., 2018). Other TCMs, such as Wuweiwan (WMW Meiwan, WMW), reduce CAC (colitis and colorectal cancer) by regulating the balance between “tumour promoting bacteria” and “carcinoma suppressing bacteria” and the NF-kB/IL-6/STAT3 pathways (Jiang et al., 2020). Gegen Qinlian Decoction (GQD) can increase the content of short-chain fatty acid producing bacteria, such as *Ruminococcus*, thereby increasing the level of fecal SCFA and reducing diarrhoea in piglets (Liu et al., 2019a). Additionally, a series of studies have proven that drug-flora-metabolites are an important mechanistic axis in the treatment of diseases.

As a traditional Chinese medicine that is commonly used to treat angina pectoris and ischemic stroke, TXL was also shown to significantly improve the stability of atherosclerotic plaques in our study. However, to date, whether the relevant effects of TXL are related to the intestinal flora has not been reported. Our study found that Tongxinluo has a significant effect on the intestinal flora of rabbits. Intervention with TXL reversed the changes of the abundance of some intestinal bacteria in rabbits induced by HCB. For example, TXL intervention increased the abundance of *Alitipes*, *Campylobacter*, *Rikenella*, *indistinctus*, *viscericola*, *nordii*, and *subantarcticus* increased and decreased the abundance *Barnesiella*, *eggerthii*, and *albus*. At the same time, a variety of metabolic components in faeces, such as trans-ferulic acid, nicotinamide, DL-3-phenyllactic acid, and hydroxyphenyllactic acid also demonstrated significant differences after Tongxinluo intervention compared with those in the non-intervention group. According to previous studies, these metabolites all play important roles in improving inflammation and atherosclerosis. For example, nicotinamide, which is a form of vitamin B3, is essential for maintaining the stability of the genome and has photoprotective and anti-inflammatory effects; hydroxyphenyllactic acid can reduce ROS (reactive oxygen species) in mitochondria and neutrophils and is a natural antioxidant (Beloborodova et al., 2012). Trans-ferulic acid, a metabolite related to plaque stability, also has anti-inflammatory, anti-diabetic, anti-cancer and cardioprotective effects.

Trans-ferulic acid is the main active form of ferulic acid and is found in many traditional Chinese herbal medicines and supplements (Cai et al., 2006). Previous studies have shown that ferulic acid is inextricably linked with inflammation. Intake of FA can reduce NADPH oxidase activity, superoxide release, apoptosis and necrosis in peripheral blood mononuclear cells (Perez-Ternero et al., 2017) and can also prevent the nephrotoxicity of methotrexate by activating the Nrf2/ARE/HO-1 and PPARγ signalling pathways and inhibiting the NF-κB/NLRP3 axis (Mahmoud et al., 2019). Our study confirmed that the effect of TFA on macrophages could inhibit expression of the NLRP3 inflammasome and Caspase-1 in concentration-dependent manners. FA is also inextricably linked with the intestinal flora. On the one hand, the intestinal flora affects the production of FA. It has been reported that six bacterial isolates from human faeces can release FA from ethyl esters: *Escherichia coli* (3 strains), *Bifidobacterium lactis*, and *Lactobacillus gasseri* (two strains) (Couteau et al., 2001). On the other hand, a study of FA in rats showed that FA could reversely regulate the intestinal flora by itself. FA increases the abundance and diversity of related bacteria (Ou et al., 2016; Teixeira et al., 2018), regulates the F/B ratio (Ma et al., 2019), increases intestinal lactic acid bacteria and improves the heart function of TAC mice (Liu et al., 2019b).

In the colon, FA is generated from its parent compound via microbial cinnamic acid esterase (Couteau et al., 2001). In our study, non-targeted metabolomics detection of colonic stool showed that the abundance of TFA was significantly different after TXL intervention (Supplementary Figure S1F); however, Spearman correlation analysis showed that there was no obvious correlation between the intestinal flora and TFA. We believe that FA can be produced by a variety of bacteria, so its content in feces may be affected by multiple bacteria rather than a single strain. Ferulic acid is mainly absorbed through passive diffusion. Studies have confirmed that the form of ferulic acid available in the blood after crossing the colonic barrier is mainly the free form, and free ferulic acid is mainly transferred by passive diffusion; only a small proportion (<20%) is transferred by a carrier (Poquet et al., 2008). Therefore, in the initial stage, the concentration of FA in the blood should be consistent with its concentration in the intestines. However, the metabolism of FA is extremely fast, for example, in rats, the half-life of FA is 30 min, and in humans, the half-life of FA is 45 min (Mancuso and Santangelo, 2014). Therefore, after 8 h of fasting, the concentration of TFA in serum was not significantly changed (Supplementary Figure S1E). Therefore, the difference in TFA in serum caused by TXL intervention should be studied further to determine the pharmacokinetics of the effect. Of course, many other metabolites, such as nicotinamide, DL-3-phenyllactic acid, and hydroxyphenyllactic acid, may also produce the same effects according to previous research results, and the specific mechanisms require further study.

Fecal microbiota transplantation (FMT) is a common method used to prove the causal relationships between flora and many diseases. In future research, we plan to increase the sample size, use overall flora transplantation or single bacteria and metabolite enhancement to conduct in-depth studies to clarify specific bacterial species and differential metabolites that participate in Tongxinluo’s anti-inflammatory and stabilizing plaques effects.

## 5 Conclusion

In conclusion, TXL plays an important anti-atherosclerotic role. TXL changes the composition of the intestinal flora and intestinal metabolites, thereby inhibiting expression of NLRP3 and its downstream inflammatory factors IL-1β and IL-18 in vascular tissue, and improving the stability of plaques. This study provides new theoretical support for the role of TXL and has important clinical significance.

## Data Availability

The datasets presented in this study can be found in online repositories. The names of the repository/repositories and accession number(s) can be found below: NCBI database, accession number PRJNA779551.
