# Supplementary material for: Tongxinluo May Alleviate Inflammation and Improve the Stability of Atherosclerotic Plaques by Changing the Intestinal Flora
Source: Front Pharmacol. 2022 Apr 1;13:805266. doi: 10.3389/fphar.2022.805266 (PMC9011338; doi:10.3389/fphar.2022.805266)
Supplement: Supplementary file 1 [file DataSheet1.docx]

**Supplement figure and tables**

**Supplement figure**

**
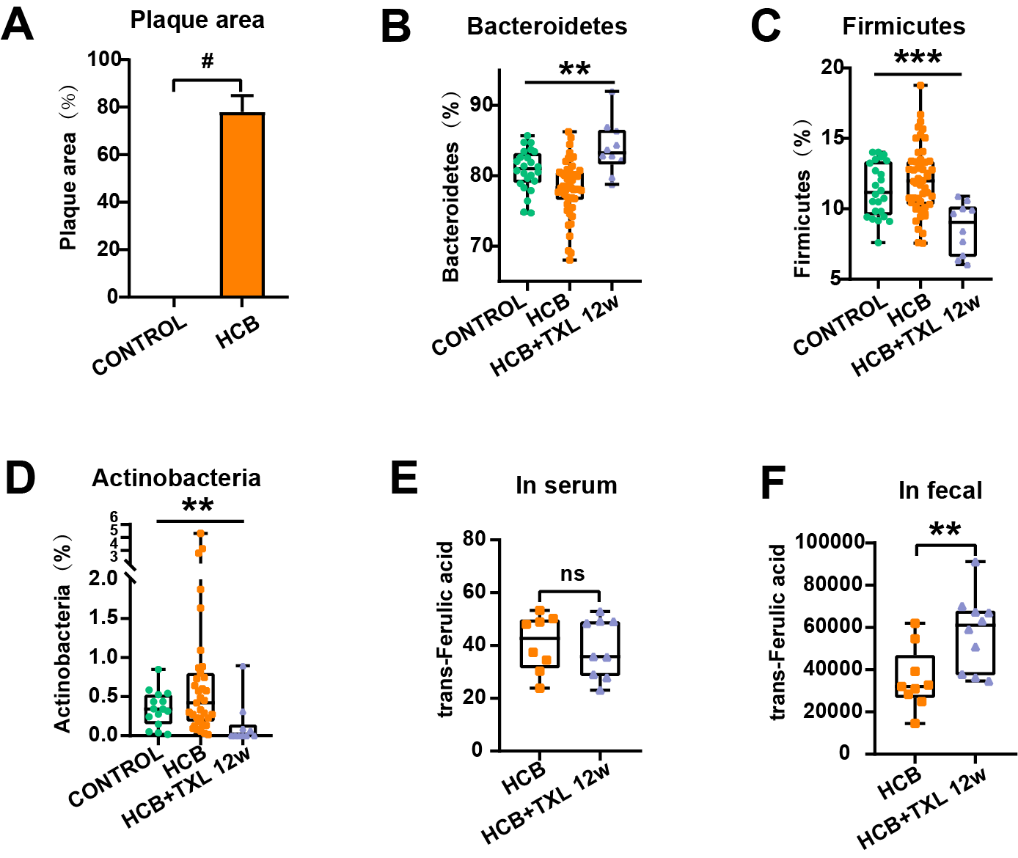
**

**Supplement figure：**(A), Count the proportion of plaque area in the aorta after the Oil Red O staining. And non-parametric test was used for statistical, data were shown as the mean ± S. E. M(H). #: p< 0.0001. (B, C, D) The relative abundance difference statistics of Bacteroidetes phylum, Firmicutes phylum and Actinobacteria phylum in CONTROL (n = 24), HCB (n = 52) and HCB+TXL 12w (n=10) groups. Data were presented as median (minimum to maximum). One-way ANOVA analysis was used for statistical. **: p<0.01; ***: p<0.001. (E, F) The changes of trans-ferulic acid in serum and in fecal of rabbits in HCB group and HCB+TXL 12w group. Data were presented as median (minimum to maximum), and non-parametric test was used for statistical. ns: no significance; **: p<0.01.

**Supplement Table 1.**

**The main of significantly changed metabolites between control group and HCB group.**

| **Metabolites** | **CONTROL** | **HCB** | **Fold change（FC）** | **Log2FC** | **up/down** | **p-value** |
| --- | --- | --- | --- | --- | --- | --- |
| **N-Hydroxy-L-valine** | 3204.487 | 24488.126 | 7.642 | 2.934 | UP | 0.0039054 |
| **S,S-Dimethyl-beta-propiothetin** | 1019669.300 | 7293122.111 | 7.152 | 2.838 | UP | 0.0000403 |
| **Tyramine** | 18395.192 | 105808.871 | 5.752 | 2.524 | UP | 0.0090242 |
| **4-Hydroxyphenylethanol** | 36008.687 | 193251.458 | 5.367 | 2.424 | UP | 0.0002824 |
| **Cholic acid** | 42956.825 | 211749.708 | 4.929 | 2.301 | UP | 0.0000017 |
| **4-(Methylnitrosamino)-1-(3-pyridyl)-1-butanol** | 18169.318 | 79989.202 | 4.402 | 2.138 | UP | 0.0003252 |
| **L-Homocystine** | 12359.106 | 46489.752 | 3.762 | 1.911 | UP | 0.0098342 |
| **NG,NG-Dimethyl-L-arginine** | 17367.651 | 59782.937 | 3.442 | 1.783 | UP | 0.0000806 |
| **Feruloylputrescine** | 44379.042 | 145640.026 | 3.282 | 1.714 | UP | 0.0000872 |
| **Cholesteryl sulfate** | 114797.537 | 375919.722 | 3.275 | 1.711 | UP | 0.0017196 |
| **L-Kynurenine** | 3553.575 | 11295.700 | 3.179 | 1.668 | UP | 0.0107778 |
| **Cephalosporin C** | 29039.953 | 84972.258 | 2.926 | 1.549 | UP | 0.0053224 |
| **2-Hydroxy-3-methylbutyric acid** | 303974.878 | 848890.789 | 2.793 | 1.482 | UP | 0.0091683 |
| **Dimethylaminopurine** | 51031.023 | 129529.711 | 2.538 | 1.344 | UP | 0.0007560 |
| **4-Hydroxy-2-quinolone** | 10672.874 | 25126.899 | 2.354 | 1.235 | UP | 0.0005213 |
| **4-Hydroxy-L-threonine** | 6478.237 | 15215.574 | 2.349 | 1.232 | UP | 0.0324325 |
| **Lotaustralin** | 7700.153 | 17922.536 | 2.328 | 1.219 | UP | 0.0000257 |
| **Peganine** | 16544.800 | 38370.089 | 2.319 | 1.214 | UP | 0.0026824 |
| **Pelletierine** | 18108.505 | 41876.662 | 2.313 | 1.209 | UP | 0.0008443 |
| **3-alpha-Mannobiose** | 114985.004 | 264884.833 | 2.304 | 1.204 | UP | 0.0012434 |
| **3b-Hydroxy-5-cholenoic acid** | 240608.983 | 512018.444 | 2.128 | 1.090 | UP | 0.0000401 |
| **Urocanic acid** | 574530.333 | 1205478.167 | 2.098 | 1.069 | UP | 0.0022355 |
| **4-Methylaminobutyrate** | 39316.104 | 81534.431 | 2.074 | 1.052 | UP | 0.0001294 |
| **N-Acetylmuramate** | 244442.058 | 490980.678 | 2.009 | 1.006 | UP | 0.0012831 |
| **Urocanate** | 95510.240 | 190776.089 | 1.997 | 0.998 | UP | 0.0027864 |
| **cis-(Homo)3-aconitate** | 23648.588 | 46566.998 | 1.969 | 0.978 | UP | 0.0000000 |
| **L-Pyroglutamic acid** | 521380.242 | 1013320.589 | 1.944 | 0.959 | UP | 0.0004096 |
| **Isoniazid** | 8848.834 | 16830.404 | 1.902 | 0.928 | UP | 0.0108909 |
| **Glycochenodeoxycholate 7-sulfate** | 8571.211 | 16224.061 | 1.893 | 0.921 | UP | 0.0001303 |
| **Octadecanal** | 114800.659 | 216569.122 | 1.886 | 0.916 | UP | 0.0094424 |
| **Glycolithocholate** | 33940.481 | 63905.142 | 1.883 | 0.913 | UP | 0.0220709 |
| **(R)-4-Phosphopantothenoyl-L-cysteine** | 11685.017 | 21782.060 | 1.864 | 0.898 | UP | 0.0000317 |
| **Phosphonoformyl-CMP** | 747.447 | 1387.329 | 1.856 | 0.892 | UP | 0.0055779 |
| **Chenodeoxycholate** | 861482.042 | 1578203.333 | 1.832 | 0.873 | UP | 0.0000593 |
| **2,2,3-Trihydroxy-3-methoxy-5,5-dicarboxybiphenyl** | 13116.140 | 24020.068 | 1.831 | 0.873 | UP | 0.0340817 |
| **3alpha,12alpha-Dihydroxy-5beta-chol-6-enoate** | 6619122.833 | 12101702.111 | 1.828 | 0.870 | UP | 0.0008149 |
| **(-)-Ureidoglycolate** | 32831.364 | 59340.030 | 1.807 | 0.854 | UP | 0.0000319 |
| **beta-Alanyl-L-arginine** | 15359.334 | 27574.509 | 1.795 | 0.844 | UP | 0.0014560 |
| **Shikimate 3-phosphate** | 5346.022 | 9559.434 | 1.788 | 0.838 | UP | 0.0000855 |
| **DL-Mandelic acid** | 482597.025 | 842288.800 | 1.745 | 0.803 | UP | 0.0007478 |
| **Urate** | 5423.571 | 9431.638 | 1.739 | 0.798 | UP | 0.0032159 |
| **2-Amino-3,7-dideoxy-D-threo-hept-6-ulosonic acid** | 10386.734 | 17924.906 | 1.726 | 0.787 | UP | 0.0236679 |
| **(4Z,7Z,10Z,13Z,16Z,19Z)-Docosahexaenoic acid** | 37447881.667 | 62098637.778 | 1.658 | 0.730 | UP | 0.0272275 |
| **dAMP** | 9463.627 | 15501.037 | 1.638 | 0.712 | UP | 0.0034257 |
| **S-(Hercyn-2-yl)-L-cysteine S-oxide** | 19174.618 | 30818.392 | 1.607 | 0.685 | UP | 0.0023981 |
| **dCTP** | 1978.183 | 3147.565 | 1.591 | 0.670 | UP | 0.0106704 |
| **Cycloserine** | 64462.736 | 94601.046 | 1.468 | 0.553 | UP | 0.0013194 |
| **2-Amino-4,5-dihydroxy-6-oxo-7-(phosphooxy)heptanoate** | 30515.301 | 22607.128 | 0.741 | -0.433 | DOWN | 0.0103226 |
| **2-Amino-3-carboxymuconate semialdehyde** | 29016.814 | 20779.231 | 0.716 | -0.482 | DOWN | 0.0156628 |
| **CDP-choline** | 8619.385 | 6161.305 | 0.715 | -0.484 | DOWN | 0.0313134 |
| **p-Coumaroylagmatine** | 27510.572 | 19446.150 | 0.707 | -0.501 | DOWN | 0.0221722 |
| **Linoleic acid** | 220644.767 | 155722.944 | 0.706 | -0.503 | DOWN | 0.0091210 |
| **(-)-Medicarpin** | 460218.475 | 296640.133 | 0.645 | -0.634 | DOWN | 0.0351809 |
| **L-2-Aminoadipate adenylate** | 20471.416 | 12846.492 | 0.628 | -0.672 | DOWN | 0.0265040 |
| **10-Formyldihydrofolate** | 38851.520 | 23769.224 | 0.612 | -0.709 | DOWN | 0.0002014 |
| **Ile-Leu** | 228972.758 | 137581.432 | 0.601 | -0.735 | DOWN | 0.0016610 |
| **Glutathione** | 1741214.417 | 1019702.300 | 0.586 | -0.772 | DOWN | 0.0002970 |
| **3-Ketosucrose** | 69630.786 | 40124.147 | 0.576 | -0.795 | DOWN | 0.0238242 |
| **p-Hydroxyphenylacetylglycine** | 14482.715 | 8287.417 | 0.572 | -0.805 | DOWN | 0.0293434 |
| **Dihydrozeatin riboside monophosphate** | 141133.303 | 79198.488 | 0.561 | -0.834 | DOWN | 0.0001804 |
| **L-Sorbose** | 988193.925 | 530805.600 | 0.537 | -0.897 | DOWN | 0.0000701 |
| **D-Maltose** | 201291.963 | 107618.712 | 0.535 | -0.903 | DOWN | 0.0047946 |
| **S-Adenosyl-L-homocysteine** | 51913.907 | 26667.304 | 0.514 | -0.961 | DOWN | 0.0000601 |
| **5,10-Methenyltetrahydrofolate** | 151523.182 | 74921.832 | 0.494 | -1.016 | DOWN | 0.0012407 |
| **D-Lactose** | 2236727.667 | 1009016.500 | 0.451 | -1.148 | DOWN | 0.0002465 |
| **1-Palmitoyl-sn-glycero-3-phosphocholine** | 861294.667 | 384045.033 | 0.446 | -1.165 | DOWN | 0.0001564 |
| **N-Methylpelletierine** | 226939.992 | 100248.307 | 0.442 | -1.179 | DOWN | 0.0000194 |
| **Cellobiose** | 56715.323 | 24593.427 | 0.434 | -1.205 | DOWN | 0.0000663 |
| **Maltotriose** | 236779.375 | 101911.124 | 0.430 | -1.216 | DOWN | 0.0001223 |
| **Isoniazid alpha-ketoglutaric acid** | 262978.913 | 109721.157 | 0.417 | -1.261 | DOWN | 0.0171249 |
| **Cellohexaose** | 6931.066 | 2838.200 | 0.409 | -1.288 | DOWN | 0.0001390 |
| **D-Glucose 6-sulfate** | 203220.193 | 81906.832 | 0.403 | -1.311 | DOWN | 0.0000745 |
| **Galactinol** | 317457.975 | 122540.874 | 0.386 | -1.373 | DOWN | 0.0003292 |
| **2-Quinolinecarboxylic acid** | 51943.967 | 19785.069 | 0.381 | -1.393 | DOWN | 0.0022895 |
| **D-Galactarate** | 680292.017 | 168888.201 | 0.248 | -2.010 | DOWN | 0.0173150 |
| **Adenosine 2,3-cyclic monophosphate** | 44043.414 | 8272.234 | 0.188 | -2.413 | DOWN | 0.0279932 |

**Supplement Table 2.**

**The main of significantly changed metabolites between HCB group and HCB+TXL 12w group.**

| **Metabolites** | **HCB** | **HCB+TXL 12w** | **Fold change（FC）** | **Log2FC** | **up/down** | **p-value** |
| --- | --- | --- | --- | --- | --- | --- |
| **(2S)-2-{[1-(R)-Carboxyethyl]amino}**  **pentanoate** | 6522.365 | 21218.031 | 3.253 | 1.702 | UP | 0.0120486 |
| **5-L-Glutamyl-taurine** | 2929.810 | 8404.278 | 2.869 | 1.520 | UP | 0.0222800 |
| **1-Palmitoyl-2-hydroxy-sn-glycero-3-phosphoethanolamine** | 117563.324 | 311897.057 | 2.653 | 1.408 | UP | 0.0421996 |
| **N-Acetyl-L-glutamate** | 6371.036 | 16137.489 | 2.533 | 1.341 | UP | 0.0072381 |
| **1-Myristoyl-sn-glycero-3-phosphocholine** | 29706.068 | 70341.345 | 2.368 | 1.244 | UP | 0.0097356 |
| **Hydroxyphenyllactic acid** | 64865.568 | 153087.337 | 2.360 | 1.239 | UP | 0.0474707 |
| **Nitrilotriacetic acid** | 11502.070 | 23764.771 | 2.066 | 1.047 | UP | 0.0234415 |
| **Pyrimidine 5-nucleotide** | 297126.148 | 587251.669 | 1.976 | 0.983 | UP | 0.0362373 |
| **cis-(Homo)2-aconitate** | 3807.326 | 7254.243 | 1.905 | 0.930 | UP | 0.0310254 |
| **7,8-Didemethyl-8-hydroxy**  **-5-deazariboflavin** | 3810.895 | 7233.701 | 1.898 | 0.925 | UP | 0.0239353 |
| **1,2-Dihydrophthalic acid** | 7362.389 | 13931.178 | 1.892 | 0.920 | UP | 0.0186602 |
| **L-2-Aminoadipate adenylate** | 12846.492 | 23881.982 | 1.859 | 0.895 | UP | 0.0342338 |
| **16-Feruloyloxypalmitate** | 245402.211 | 445134.847 | 1.814 | 0.859 | UP | 0.0079635 |
| **1-Palmitoyl-sn-glycero-3-phosphocholine** | 384045.033 | 690936.518 | 1.799 | 0.847 | UP | 0.0263227 |
| **3-Deoxy-D-manno-octulosonate** | 22887.609 | 38355.715 | 1.676 | 0.745 | UP | 0.0429601 |
| **L-Threonylcarbamoyladenylate** | 12113.545 | 18650.869 | 1.540 | 0.623 | UP | 0.0025209 |
| **N-Methylaniline** | 13738.532 | 21132.719 | 1.538 | 0.621 | UP | 0.0425372 |
| **L-Cystine** | 6829.511 | 10438.103 | 1.528 | 0.612 | UP | 0.0481139 |
| **Stachyose** | 90220.218 | 125619.639 | 1.392 | 0.478 | UP | 0.0067305 |
| **4-Hydroxy-2-quinolone** | 17934.452 | 24675.283 | 1.376 | 0.460 | UP | 0.0260138 |
| **2-Methylpropanamine** | 22731.042 | 28849.626 | 1.269 | 0.344 | UP | 0.0470720 |
| **6-Tuliposide B** | 10410.647 | 7464.742 | 0.717 | -0.480 | DOWN | 0.0038657 |
| **Chenodeoxycholate** | 13417477.11 | 9464900.040 | 0.705 | -0.503 | DOWN | 0.0381072 |
| **N-Acetylneuraminate** | 121949.487 | 83555.062 | 0.685 | -0.545 | DOWN | 0.0073838 |
| **Erucic acid** | 274751.011 | 186011.400 | 0.677 | -0.563 | DOWN | 0.0403429 |
| **Cytidine** | 97003.288 | 64434.067 | 0.664 | -0.590 | DOWN | 0.0099735 |
| **3-Oxo-5beta-cholanate** | 263899.219 | 159623.974 | 0.605 | -0.725 | DOWN | 0.0126753 |

**Supplement Table 3.**

**Blood lipids and serum inflammatory factors concentration in CONTROL, HCB and HCB+TXL12w groups.**

|  | **CONTROL** | **HCB** | **HCB+TXL 12w** |
| --- | --- | --- | --- |
| **TC, mmol/l****（SEM）** | 0.9322（0.18） | 28.3522（3.62） | 24.8523（4.13） |
| **TG, mmol/l （SEM）** | 0.6450（0.16） | 2.6435（0.60） | 2.3610（0.63） |
| **HDL-C, mmol/l（SEM）** | 0.6547（0.09） | 0.6510（0.10） | 0.6751（0.09） |
| **LDL-C, mmol/l （SEM）** | 0.1599（0.04） | 10.5729（1.60） | 8.5241（1.38） |
| **IL-1β, pg/ml（SEM）** | 33.0294(1.55) | 43.3538(1.82) | 44.0149(0.93) |
| **TNF-α, pg/ml（SEM）** | 193.2904(10.33) | 246.4461(8.14) | 192.8922(14.72) |

CONTROL: normal diet (n=12); HCB: high cholesterol diet (n=8); HCB+TXL 12w: high cholesterol diet and treated with TXL for 12 weeks (n=10). w: week. TC, total cholesterol; TG, triglyceride; HDL-C, high density lipoprotein cholesterol; LDL-C, low density lipoprotein cholesterol; IL-1β: interleukin-1β; TNF-α: tumor necrosis factor-α. SEM: Standard Error of Mean.
